# Coevolution of Vertex Weights Resolves Social Dilemma in Spatial Networks

**DOI:** 10.1038/s41598-017-15603-2

**Published:** 2017-11-09

**Authors:** Chen Shen, Chen Chu, Hao Guo, Lei Shi, Jiangyan Duan

**Affiliations:** 1 School of Statistics and Mathematics, Yunnan University of Finance and Economics, Kunming, Yunnan 650221 China; 2 School of Life Science, Shanxi Normal University, Linfen, Shanxi 041004 China; 3 Shanghai Lixin University of Accounting and Finance, Shanghai, 201209 China

## Abstract

In realistic social system, the role or influence of each individual varies and adaptively changes in time in the population. Inspired by this fact, we thus consider a new coevolution setup of game strategy and vertex weight on a square lattice. In detail, we model the structured population on a square lattice, on which the role or influence of each individual is depicted by vertex weight, and the prisoner’s dilemma game has been applied to describe the social dilemma of pairwise interactions of players. Through numerical simulation, we conclude that our coevolution setup can promote the evolution of cooperation effectively. Especially, there exists a moderate value of *δ* for each *ε* that can warrant an optimal resolution of social dilemma. For a further understanding of these results, we find that intermediate value of *δ* enables the strongest heterogeneous distribution of vertex weight. We hope our coevolution setup of vertex weight will provide new insight for the future research.

## Introduction

Cooperation is ubiquitous ranging from bacteria to animals as well as human societies^1–3^. But, how to interpret the evolution of cooperation among selfish individuals represents one of the most interesting challenges in nature and social sciences and has attracted much attention across a myriad of disciplines, such as mathematics, evolutionary biology, statistical physics, to name but a few^4,5^. Evolutionary game theory has provided a mathematical framework for addressing this intriguing challenge^6–8^. Particularly, the prisoner’s dilemma game (PDG), served as a paradigm for expressing a social poverty in the case of pairwise interactions, has been used frequently to study such an overarching issue in both theoretical and experimental literatures^9,10^. In its basic version, two agents are asked to simultaneously make a choice between cooperation (C) and defection (D). They both receive *R* (*P*) if mutual cooperation (mutual defection). If one player cooperates while the other defects, the latter can get a temptation to defect *T*, and the former receives sucker’s payoff (*S*). The ranking of these payoff are ordered as $T > P > R > S$ so that defection is the best choice regardless of the opponent’s choice, which ultimately results in social dilemma^11^.

Up till now, several mechanisms have been proposed to resolve this evolutionary conundrum^12–23^. Nowak attributed all these mechanisms to five rules for the promotion of cooperation named direct reciprocity, indirect reciprocity, kin selection, group selection, and spatial reciprocity^24^. An important seminal research that inspired much more following works was the introduction of spatial structure by Nowak and May^25^, which enabled cooperators to form compact clusters on the structured network to protect the interior from being exploited by defectors. In line with this pioneering work, various types of spatial topology have been introduced into this scope to investigate the evolution of cooperation. For example, complex network, such as BA scale free network^26^, ER random graph^27^, small-word network^28^ as well as multilayer coupling network^29^, has been proved to be an effective way for maintaining cooperation. In addition, different factors have also been considered in structured population for exploring its impact on the evolution of cooperation, for example, age structure^30,31^, reputation^32,33^, memory^34,35^, voluntary participation^36,37^, social diversity^38,39^, to name but a few.

More recently, coevolution scenarios, served as the catalyst for the evolution of cooperation, have received much attention. Whereby, strategies and some other properties, such as the links between players^40,41^, the teaching ability of players^42^, the motion of players^43^, and network structure, synchronously evolve, for a comprehensive understanding referring to refs^44,45^. In spite of reaching prominent progress, the role of players’ weight receives little attention, which seems more widespread in real society. Due to the discrepancy of people, each individual may exhibit the heterogeneity of social statues or social influence in the population, which may adaptively change in time. In fact, players that possess higher social influence have larger fitness than players with lower social influence. With this point, in this paper, we investigate how cooperation fares by studying the coevolution of vertex weight and strategy within the prisoner’s dilemma game. We find that, although all players have the same social influence initially, our coevolution setup quickly results in a normal distribution of players’ social influence, which in turn facilitates the evolution of cooperation. In the remainder of this paper, we first describe our modified model; later present our simulation results, and summarize our conclusions.

## Results

**Figure 1:**
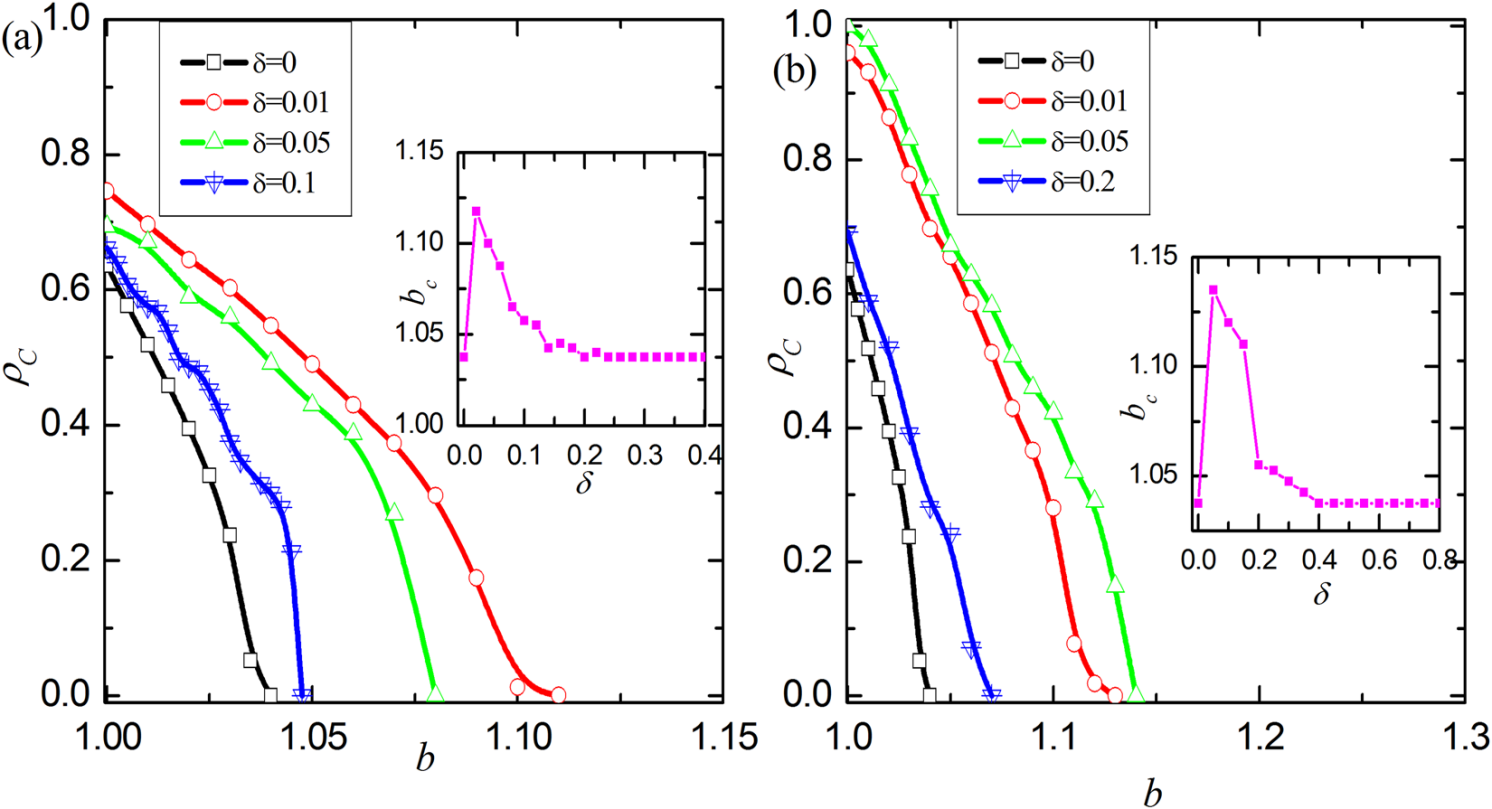
The fraction of cooperation ${\rho }_{{\rm{c}}}$ in dependence on the temptation to defect *b* for different values of $\delta$. (**a**) and (**b**) show the results of the case $\varepsilon =0.4$ and $\varepsilon =0.8$. For the traditional game, i.e. $\delta =0$, cooperation vanishes for small temptations. For positive $\delta$ the fraction of cooperation could be greatly enhanced and there is an optimal $\delta$ for which cooperation is enhanced. The inset show the relationship between the threshold ${b}_{c}$, where cooperation dies out, and $\delta$.

We start by examining the influence of parameter $\delta$ on the evolution of cooperation. Figure 1 features how the fraction of cooperation varies as a function of the temptation to defect *b* for different values of $\delta$. In particular, $\delta =0$ returns to the traditional version, where cooperation dies out soon for very small *b*. While $\delta  > 0$ introduces the heterogeneity of vertex weight, it seems to promote the evolution of cooperation from Fig. 1. However, the level of cooperation will gradually decline, which indicates that moderate $\delta$ provide the best evolutionary environment: cooperators can not only survive over a larger interval of *b*, but even dominate the spatial grid. This observation is universal for different values of $\varepsilon$. To give a broad description about the influence of $\delta$. We define one critical threshold ${b}_{c}$, which marks the vanishment of cooperation with the smallest *b*. The insets of Fig. 1 shows the relationship between threshold ${b}_{c}$ and $\delta$. Similar to the evolution of cooperation, there is an coherent resonance (i.e. a bell-shape curve): middle $\delta$ generates the largest ${b}_{c}$. The larger the value of $\varepsilon$, the more obvious this trend. All together, these observations suggest that such a simple coevolution mechanism about vertex weight and strategy not only enhances the survival of cooperation, but also guarantees the best optimal level.

**Figure 2:**
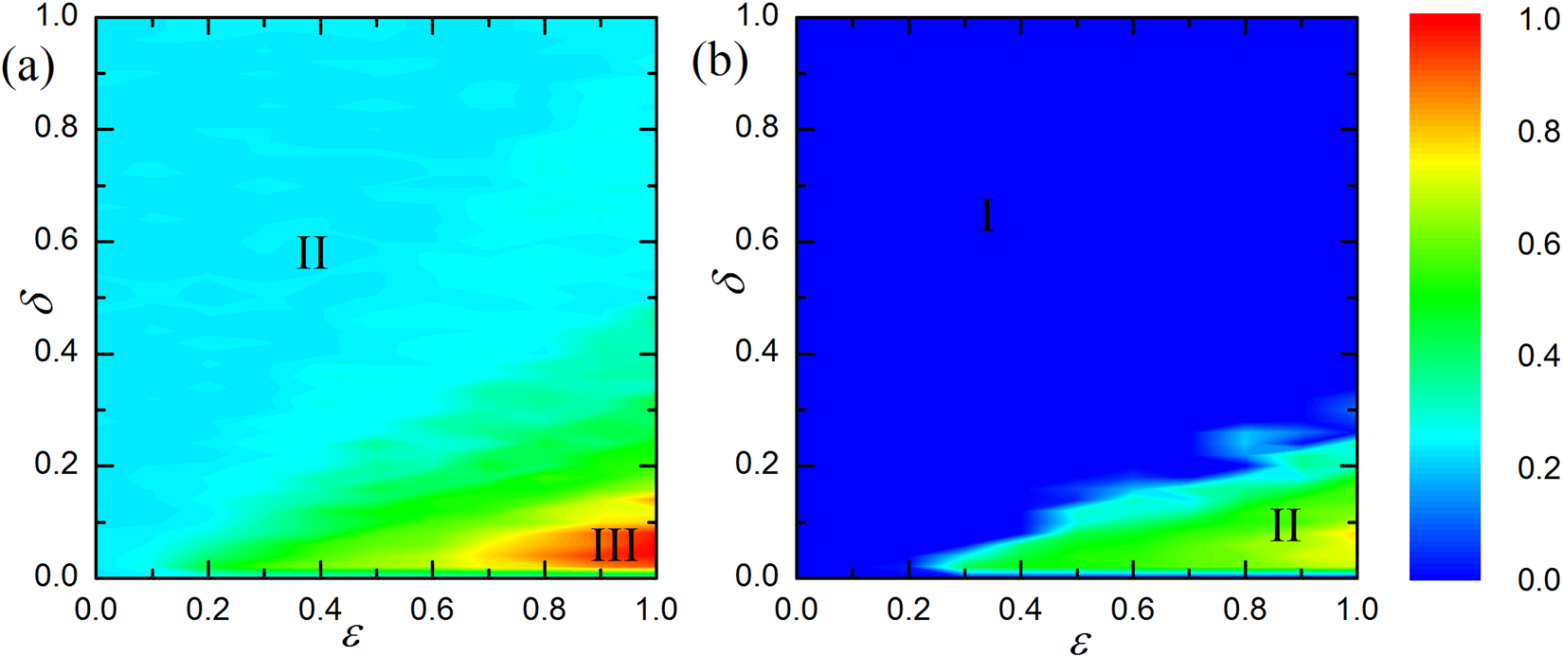
Color-code (see bar on the right) fraction of cooperation on the $\delta -\varepsilon$ parameter plane for *b* = 1.03 (Fig. 2(a)) and *b* = 1.06 (Fig. 2(b)). Both panels show that the optimal value, where cooperation is best promoted, is about 0.05 irrespective of which temptation to defect *b* applies.

To give a complete understanding of such a coevolution mechanism. Figure 2 presents the color map encoding the fraction of cooperation ${\rho }_{c}$ on the $\delta -\varepsilon$ parameter plane for different values of *b*. Interestingly, the whole plane is divided into three phases: full defection phase (phase I), well-mixed phase for cooperation and defection (phase II) and full cooperation phase (phase III). As $\varepsilon$ increases, the survival of cooperators will become relatively easy. In particular, for small and middle $\delta$, increament of $\varepsilon$ enables system to produce various phase transitions, from phase II to phase III. That is to say, increasing $\varepsilon$ induces stronger heterogeneity, which plays a crucial role in promoting cooperation as shown in refs^38–40,46^. While for the impact of $\delta$, it is evident that with strong heterogeneity, middle $\delta$ produces an optimal environment for cooperation (i.e. phase III). However, with continuous enhancement of $\delta$, the maintenance of cooperation remains a challenge. For larger *b* (see Fig. 2(b)), the territory of cooperation will become smaller yet the higher level of cooperation is robust. As such, the proposal of coevolution mechanism could guarantee beneficial environment of cooperation.

**Figure 3:**
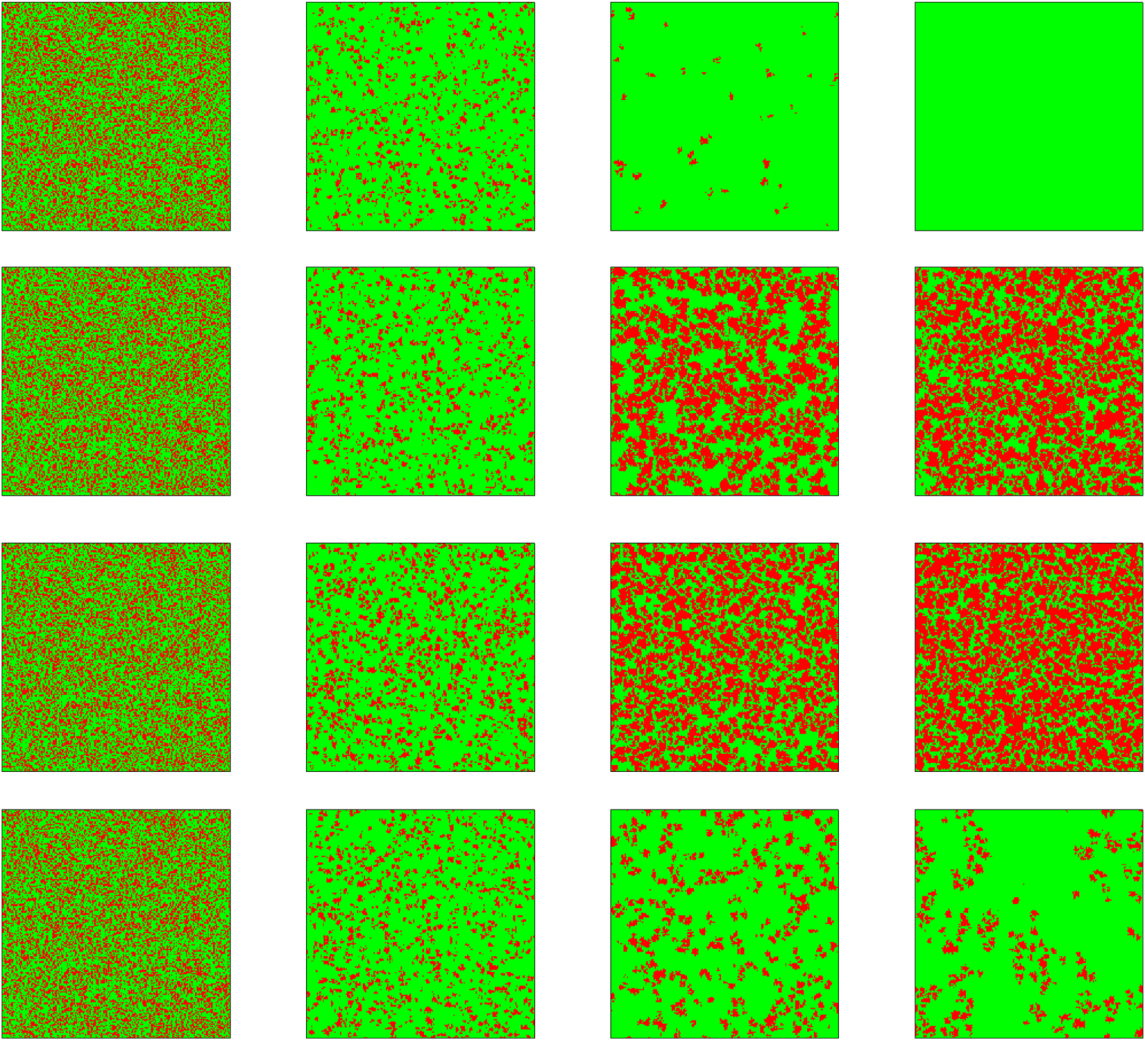
Typical snapshots of the distribution of strategy in step 0, 10, 100, 30000. All results are obtained for $\varepsilon =0.8$ and $b=1.06$. From top to bottom, $\delta$ are equal to 0, 0.01, 0.05, and 0.2 respectively. Cooperators and defectors are marked by red and green.

Subsequently, it is instructive to give an understanding why this mechanism resolves the social dilemma. Figure 3 features the spatial distribution of cooperators and defectors for different steps. From top to bottom, the value of $\delta$ are equal to 0, 0.01, 0.05 and 0.2, respectively. Initially, cooperators and defectors are randomly distributed on the square lattice, defectors get more benefits from cooperators and thus cooperators cannot form compact clusters to resist the invasion of defectors (see the second column) and cooperation will vanish soon. However, at the second panel, we can see that the cooperators will suffer the attack of defectors and they can survive by small *C* groups or patches. It is worth mentioning that at this moment because there are rare cooperators left to be exploited by defectors, the advantage of defectors is greatly reduced. Soon, cooperators recover the lost ground till saturated stable state, where *C* clusters are much more compact and the distance between them is much smaller than the size of clusters. There remains smaller space for cooperation. As $\delta$ continues to increase (the third panel), the formation and expandation of compact C clusters becomes more obvious, which supresses the exploitaion of defection. As expected, for enough large $\delta$, though some C clusters survive, they become diluted, thus there is no sufficient protection for cooperators’ territory. Nevertheless, it is clear that the evolution of cooperation is closely related to C clusters.

**Figure 4:**
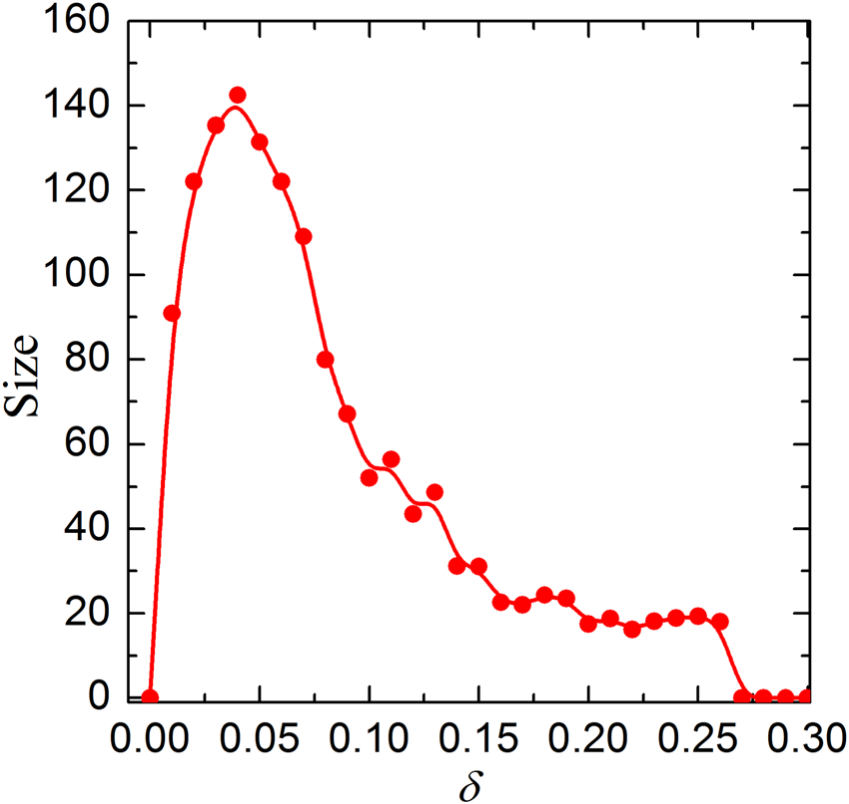
Stationary average size of cooperators cluster (${S}_{c}$) for a 200*200 square lattice with $\varepsilon =0.8$ and $b=1.06$. Obviously, optimal value of $\delta$ guarantees largest ${S}_{c}$, which too small or too large $\delta$ reduces ${S}_{c}$.

**Figure 5:**
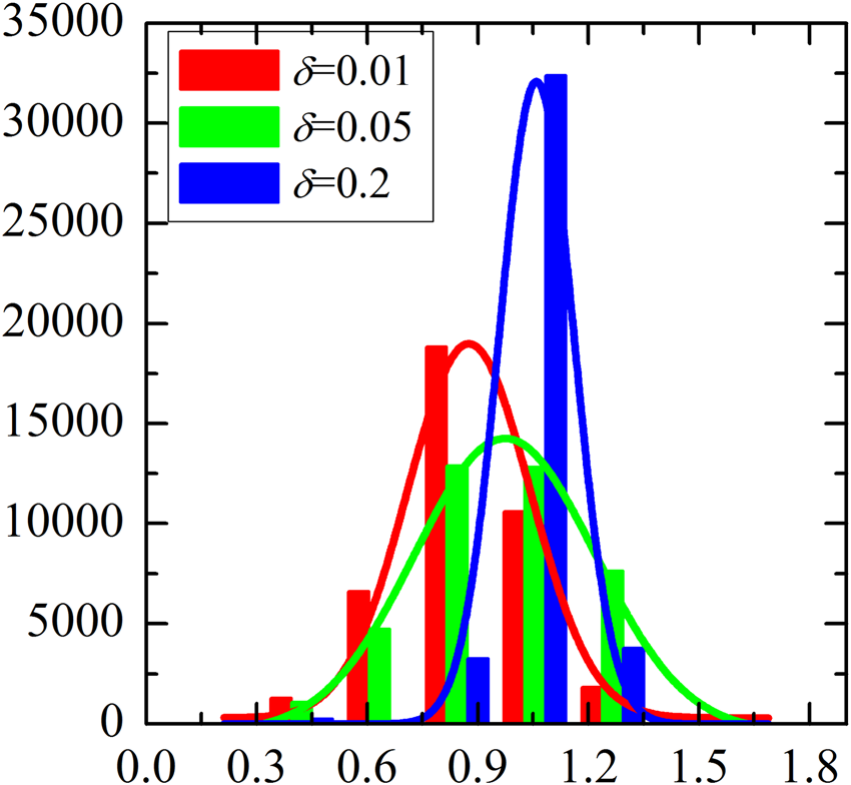
The histogram of the distribution of vertex weight under coevolution mechanism. All the results are obtained for $\varepsilon =0.8$ and $b=1.06$. The fitting line indicates that a normal distribution emerges irrespective of which $\delta$ applies, and the ranking of the variance of three cases is $Va{r}_{\delta =0.05} > Va{r}_{\delta =0.01} > Va{r}_{\delta =0.2}$.

Along this line, we next give some quantitative descriptions about C culsters in this coevolution mechanism. Figure 4 shows average size of C clusters (${S}_{c}$) as a function of $\delta$. It is obvious that average C cluster size first increases with $\delta$, reaches its maximum value at $\delta \approx 0.05$ and then decreases again. That is to say, small $\delta$ only enables limited cooperators survive and the formed C clusters is relatively small. With $\delta$ increases, the size of C clusters will fast expand. However, as $\delta$ continuously increases, ${S}_{c}$ will decline, which means that the too large $\delta$ could not generate benefical ground for ${S}_{c}$ even if there are still some clusters, thus these clusters are isolated cooperator, which can not effectively resist the explortation of defection. Thus, it is easy to understand why the average size of C clusters will become zero again. Except for quantitative descriptions about C culsters, here it is instructive to further examine the potential reason of these changes. Figure 5 shows the steady distribution of vertex weight for different values of $\delta$. It is clear that the vertex weight is no longer a single value, but rather a set of data that is normally distributed approximately no matter what value of $\delta$ is. At the same time, we can also observe that the variance of vertex weight is greatly different and the order of the variance is $Va{r}_{\delta =0.05} > Va{r}_{\delta =0.01} > Va{r}_{\delta =0.2}$. In fact, we also calculate the accurate variance of these cases: $Va{r}_{\delta =0.01}$ = 0.17, $Va{r}_{\delta =0.05}=0.22$, and $Va{r}_{\delta =0.2}=0.07$, which is consitent with the results that we obtained intuitively. These results attest to the fact that large enough values of $\delta$ will lead to a heterogeneous distribution of the vertex weight, but at the same time reduce the degree of heterogeneity compared with the moderate $\delta$, since too large value of $\delta$ leads to the difference of vertex weight becoming tiny.

## Conclusion and Discussion

To conclude, motivated by the realistic situation: individual’s social influence or social statue (hereby, denoted by vertex weight) adaptively change according to their social performance, we have explored the effect of evolutionary game based on the vertex weight and strategy on the evolution of cooperation. Through numerical simulation, we find that our coevolution setup can promote the evolution of cooperation effectively, besides, moderate value of $\delta$ can provide best environment for cooperators to survive and even domain. While these observations can be attributed to the heterogeneous distribution of player’s influence, the stronger the heterogeneity is, the higher the level of cooperation. As is shown in Fig. 5, too large or too small $\delta$ will weaken the degree of players’ heterogeneity, which will lead to the deterioration of cooperation-facilitative effect. The aforementioned observation, in a sense, is similar to refs^30,39^. In this sense, we can conclude that heterogeneity can explain the cooperation-promotion phenomenon, but is insufficient to explain the promotion effect for moderate $\delta$.

The above results can help us construct a comprehensive understanding of the role of vertex weight on the evolution of cooperation under a simple framework of co-evolution model. It has been verified that different network structures have a significant impact on the evolution of cooperation. To test our model on different topologies will become more interesting in the future. Besides, interdependent networks, where seemingly irrelevant changes in one network can have catastrophic and unexpected consequence in another network, have become a hot topic in recent years. How to apply our work to interdependent networks is another interesting issue that deserves our great attention in the future.

## Methods

Here, we consider the weak prisoner’s dilemma game with the normalized payoff matrix,

1 $$A=(\begin{array}{cc}1 & 0\\ b & 0\end{array}),$$

where the parameter $b(1 < b < 2)$ denotes the temptation to defect and ensures the proper payoff ranking. It is worth mentioning that, although we don’t choose the classical PD game, the results are accordant. Each player is designed either as a cooperator ${s}_{x}=C$ or defector ${s}_{x}=D$ with equal probability. With regard to the interaction network, we choose the regular square lattice with four nearest neighbors of size $L\ast L$. Vertex weight (social influence of players) is introduced into the model in the following way: each player, at the beginning, is assigned the same social influence ${w}_{x}=1$, which, however will adaptively change in accordance with the interaction.

At each time step, a random selected player $x$ first acquires his payoff ${p}_{x}$ by playing the game with his direct neighbors. Second, the payoffs ${p}_{y}$ of all the neighbors of player $x$ can be obtained in the same way. Following ref.^47^. we can define the environment as follows:

2 $$\bar{p}=\frac{{\sum }_{y=1}^{{k}_{x}}{p}_{y}}{{k}_{x}},$$

where the sum runs over all the neighbors of player *x*, and ${k}_{x}$ denotes the degree of player *x*. Then we can calculate the fitness of player *x* in the following expression:

3 $${F}_{x}={w}_{x}\ast {p}_{x}.$$

The vertex weight ${w}_{x}$ evolves with player’s performance: if the payoff of player $x$ is larger than the environment, the vertex weight increases $\delta$ as the reward, otherwise decreases $\delta$ as the punishment, which can be described as

4 $$\{\begin{array}{c}{w}_{x}={w}_{x}+\delta ,\,{p}_{x} > \bar{p}\\ \,{w}_{x}={w}_{x},\,\,\,\,\,\,\,{p}_{x}=\bar{p}\\ {w}_{x}={w}_{x}-\delta ,\,{p}_{x} < \bar{p}\end{array}.$$

Besides, we assume that the range of vertex weight is the interval $[1-{\rm{\varepsilon }},1+{\rm{\varepsilon }}]\,$. Obviously, when $\varepsilon =0$ or $\delta =0$, it will turn to the traditional version, while ${\rm{\varepsilon }}\ne 0$ or ${\rm{\delta }}\ne 0$ incorporates the heterogeneity case. Following ref.^42^. the evolution of ${w}_{x}$ is stopped for all players as soon as one ${w}_{x}$ reaches their maximum value.

When the focal player $x$ updates his strategy, it will pick up randomly one neighbor $y$, who also gets his fitness ${F}_{y}$ in the same way, and decides whether to adopt the strategy of player $y$ with the following probability, that is,

5 $$w=\,\frac{1}{1+\exp ([({F}_{x}-{F}_{y})/K])},$$

where *K* denotes the amplitude of noise or its inverse the so-called intensity of selection. In this paper, we fix the value of *K* to be *K* = 0.1^48,49^.

Simulation results were carried out on a 200*200 square lattice. The key quantity of the fraction of cooperation ${\rho }_{{\rm{c}}}$ was determined within the last $5\times {10}^{3}$ full Monte Carlo simulation over the total $3\times {10}^{4}$ steps. Moreover, each data were averaged over up to 10 independent runs for each set of parameter values in order to assure suitable accuracy. It is worth stating that we have found qualitative results unchanged if we give the initial state (One half of the system is C, and the other area is D).
